# Airways flat angioma misdiagnosed as difficult asthma in an adolescent

**DOI:** 10.1016/j.rmcr.2020.101323

**Published:** 2020-12-14

**Authors:** Alessandro Bodini, Laura Tenero, Luca Pecoraro, Chiara Salvottini, Michele Piazza, Giorgio Piacentini

**Affiliations:** a Department of Surgical Sciences, Dentistry, Gynecology and Pediatrics, Pediatric Division, University of Verona, Verona, Italy; b Department of Medicine, University of Verona, Verona, Italy; c Pediatric Clinic, ASST Mantova, Mantova, Italy

**Keywords:** Airways flat angioma, Bronchoscopy, Difficult asthma, Adolescent, CT, computed tomography, MRI, magnetic resonance imaging

## Abstract

A 15 years-old boy came to our attention with a diagnosis of poorly controlled asthma. This case required thorough investigations: CT scan imaging revealed a flat angioma extending from the carina to the left main bronchus. Rigid bronchoscopy confirmed the presence of an angioma showing widespread mucosal diffusion involving most of the posterior tracheal wall and main bronchi, on the left side. We present this case report and these images to readers seeking for other experiences in the diagnosis of wide superficial bronchial angioma in pediatric age.

## 1 Background

Congenital malformations of the bronchial tree can be associated to symptoms like coughing, wheezing, and dyspnoea. For this reason, these congenital malformations have to be considered in the differential diagnosis of asthma in pediatric age [1]. Moreover, several diseases can mimic asthma, such as structural airway abnormalities, intrabronchial obstruction, aspiration, gastroesophageal reflux with/without recurrent micro-aspiration, cystic fibrosis, ciliary dyskinesia, immune dysfunction, bronchiectasis, pulmonary edema, lung disease of prematurity, and bronchiolitis obliterans syndrome [2]. This article describes a rare case of flat bronchial angioma, misdiagnosed as a difficult asthma in an adolescent.

## 2 Case presentation

A 15-year-old boy came to our attention with a diagnosis of poorly controlled asthma. The patient came to the attention of a Pediatric Allergy Clinic only at the age of 12 and was diagnosed with asthma. He had been treated since then with inhaled-β2-agonists and steroids with poor results. No significant bronchial reversibility was ever observed at spirometry.

**Fig. 1:**
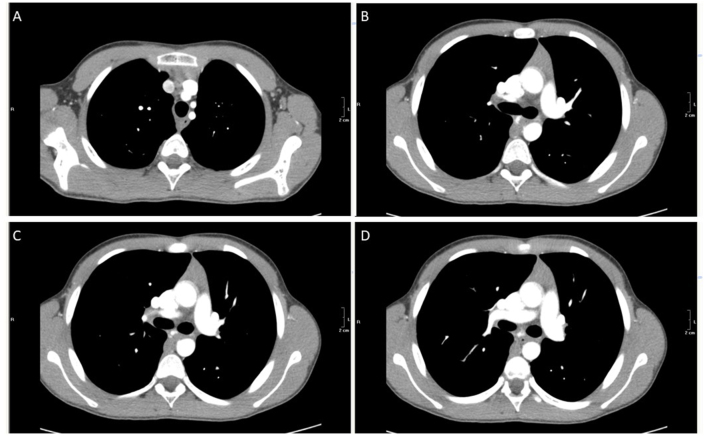
CT scan showing an area of abnormal contrast enhancement extending from the carina to the main bronchus, consistent with an angioma.

**Fig. 2:**
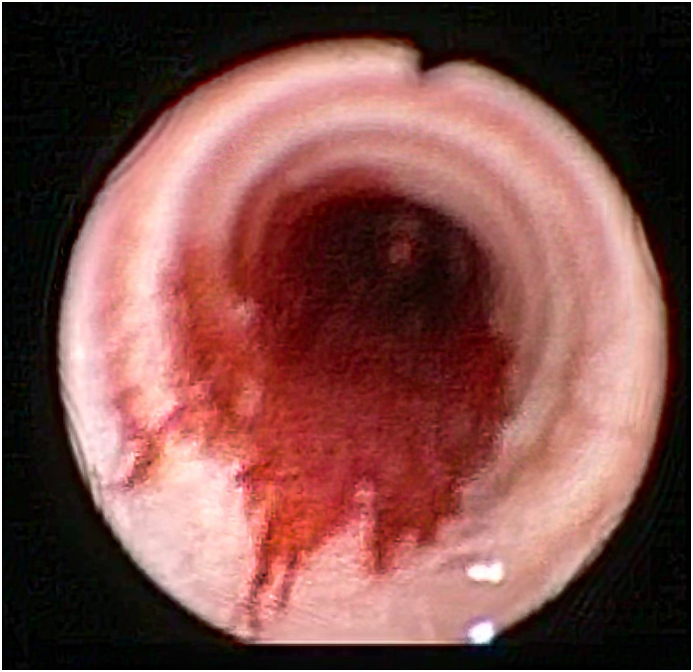
Bronchoscopic image showing a wide superficial angioma at the level of the carina.

This case of poorly controlled asthma required thorough investigations and CT scan imaging was performed, revealing a flat angioma extending from the carina to the left bronchus (Fig. 1A–D pictures). In particular, an area of abnormal contrast (Ultravist 370) enhancement was observed at the level of the carina wall, which was consistent with an angioma supplied by the right upper lobe pulmonary artery draining into the azygos vein. Therefore, rigid bronchoscopy under general anaesthesia was performed: the angioma showed widespread mucosal diffusion with flat vascular features, net margins, involving most of the posterior tracheal wall and main bronchi, on the left side (Fig. 2, video on supplementary material).

The following is/are the supplementary data related to this article:

Multimedia component 1

## 3 Discussion and conclusion

As reported, flat bronchial angiomas represent a rare cause of congenital malformations and have to be considered in the differential diagnosis of pediatric asthma [1]. Airways angiomas are usually diagnosed through bronchoscopy performed for persistent cough, stridor or other respiratory symptoms [3]. The diagnosis of airway flat angioma is often made during childhood [3]. This case report shows some peculiarities. First, the diagnosis of asthma was made even if no significant bronco-reversibility was ever observed at spirometry. Second, the treatment with inhaled-β2-agonists and steroids was administered for three years with good compliance, but poor results in terms of symptoms and spirometric values improvement. All of these peculiarities and the presence of symptoms poorly responsive to the prescribed asthma therapy justified a full clinical reassessment to exclude that the child was wrongly labeled with the diagnosis of asthma. In particular, all possible conditions which might mimic asthma needed to be considered [1]. Since our patient was an adolescent with symptoms unresponsive to therapy, a chest CT scan represented the optimal choice for the first step of the diagnostic work-up. With the detection of a flat angioma extending from the carina to the left bronchus, an optimal second step to deeply investigate the features of this congenital malformation was represented by rigid bronchoscopy under general anaesthesia. Specifically, the angioma showed widespread mucosal diffusion with flat vascular features, net margins, involving most of the posterior tracheal wall and main bronchi, on the left side. This anatomical conformation could explain the significant narrowing of the airway lumen due to the angioma. When an airway angioma is diagnosed, propranolol represents the gold standard treatment [4]. In fact, immediate treatment with propranolol should be initiated once a diagnosis of symptomatic airway angioma is confirmed, and cardiovascular assessment should be performed [4]. Unfortunately, it was impossible to follow-up the patient after the diagnosis, attempts to contact him were unsuccessful and therefore no specific therapy was started. The authors know that this represents a significant limitation to the article because of the lack of a significant cause-effect relationship that could explain the narrowing of the airway lumen. Anyway, the authors think that this case report and its radiologic images and bronchoscopic image can help other clinicians seeking for other experiences in the diagnosis of wide superficial bronchial angioma in pediatric age.

## Author's contributions to the study

AB and LP designed the study; MP and AB collected data; LP, and CS wrote the first draft of the manuscript; AB, LT and GP critically reviewed the manuscript for intellectual contents. All authors read and approved the final manuscript.

## Ethics

Consent has been obtained from the legal guardian.

## Funding

The authors received no financial support for the article.

## Declaration of competing interest

Authors declare that they have no conflict of interest.
